# A novel study on SARS‐COV‐2 virus associated bradycardia as a predictor of mortality‐retrospective multicenter analysis

**DOI:** 10.1002/clc.23622

**Published:** 2021-05-08

**Authors:** Sabina Kumar, Christina Arcuri, Sumanta Chaudhuri, Rahul Gupta, Mahendra Aseri, Pranav Barve, Shivang Shah

**Affiliations:** ^1^ Department of Internal Medicine Hemet Global Medical Center Hemet California USA; ^2^ Divison of Cardiology Loma Linda University School of Medicine Loma Linda California USA; ^3^ Division of Cardiology University of California Riverside School of Medicine Riverside California USA

**Keywords:** bradycardia, COVID‐19, mortality, SARS‐CoV2, severity, triage

## Abstract

**Background:**

SARS‐CoV2 has affected more than 73.8 million individuals. While SARS‐CoV2 is considered a predominantly respiratory virus, we report a trend of bradycardia among hospitalized patients, particularly in association with mortality.

**Methodology:**

The multi‐center retrospective analysis consisted of 1053 COVID‐19 positive patients from March to August 2020. A trend of bradycardia was noted in the study population. Absolute bradycardia and profound bradycardia was defined as a sustained heart rate < 60 BPM and < 50 BPM, respectively, on two separate occasions, a minimum of 4 h apart during hospitalization. Each bradycardic event was confirmed by two physicians and exclusion criteria included: less than 18 years old, end of life bradycardia, left AMA, or taking AV Nodal blockers. Data was fetched using a SQL program through the EMR and data was analyzed using SPSS 27.0. A logistic regression was done to study the effect of bradycardia, age, gender, and BMI on mortality in the study group.

**Results:**

24.9% patients had absolute bradycardia while 13.0% had profound bradycardia. Patients with absolute bradycardia had an odds ratio of 6.59 (95% CI [2.83–15.36]) for mortality compared with individuals with a normal HR response. The logistic regression model explained 19.6% (Nagelkerke R^2^) of variance in the mortality, correctly classified 88.6% of cases, and was statistically significant X^2^ (5)=47.10, p < .001. For each year of age > 18, the odds of dying increased 1.048 times (95% CI [1.25–5.27]).

**Conclusion:**

The incidence of absolute bradycardia was found in 24.9% of the study cohort and these individuals were found to have a significant increase in mortality.

## 1 INTRODUCTION

In December 2019, a novel betacoronavirus was discovered from a cluster of patients identified to have pneumonia due to an unknown cause. Discovered through unbiased sequencing from human airway epithelial cells, and named 2019‐nCoV, this virus had different characteristics than the previously discovered MERS‐CoV and SARS‐CoV coronaviruses.

Acute hypoxic respiratory failure is the most serious manifestation of illness leading to multi organ failure and death in these cases. While SARS‐CoV2 is considered a respiratory virus, cardiac manifestations occur more frequently than previously anticipated. These include cardiomyopathy, myocardial infarction, coronary thromboembolism, and arrhythmias among others.^1^, ^2^, ^3^

Acute cardiac injury is the most commonly reported manifestation. Mehrbod et al performed a meta‐analysis of acute cardiac injury in COVID‐19 and found the incidence to be 15%.^1^ Less commonly reported were the complications with cardiac arrhythmias, specifically tachy‐ and bradyarrhythmias. In a case series of 138 patients from Wuhan, China, 17% had an arrhythmia‐related complication.^2^ In an analysis of 700 COVID‐19 positive patients from University of Pennsylvania, they identified 8% of patients who had an arrhythmia‐related event.^15^ Gopinathannair et al sent a survey out to cardiac electrophysiologist specialist around the world, and of the 683 responses, the most common arrhythmia reported was atrial fibrillation (21%). Specifically with bradycardia, the most common arrhythmia found was sinus bradycardia (8%) and complete heart block (8%).^3^

Bradycardia in COVID‐19 appears to be an important cardiac manifestation that has a multifactorial etiology and is increasingly becoming a characteristic clinical finding in patients with COVID‐19. It has been described in isolated case reports,^4^, ^5^ a limited case series^6^ defined as relative bradycardia with heart rate (HR) < 90 beats per minute (BPM)^7^ and through a survey sent among cardiac electrophysiologists.^3^

In Southern California, there have been a multitude of patients with COVID‐19 with variable presentations. Our clinicians and cardiology colleagues noticed a significant trend of bradycardia among our hospitalized COVID‐19 patients during our initial case surge in March 2020. By definition, in cases of SIRS we expect a tachycardia response.

As our clinical observation differed from the above convention, our team decided to investigate the bradycardic response in COVID‐19.

Below, we report the first novel study to our knowledge with a sample size of over 1000 cases that comments on the prevalence of absolute bradycardia (HR < 60 BPM) and profound bradycardia (HR < 50 BPM) within a multi‐hospital COVID‐19 population with a particular association with mortality.

## 2 METHODOLOGY

**FIGURE 1:**
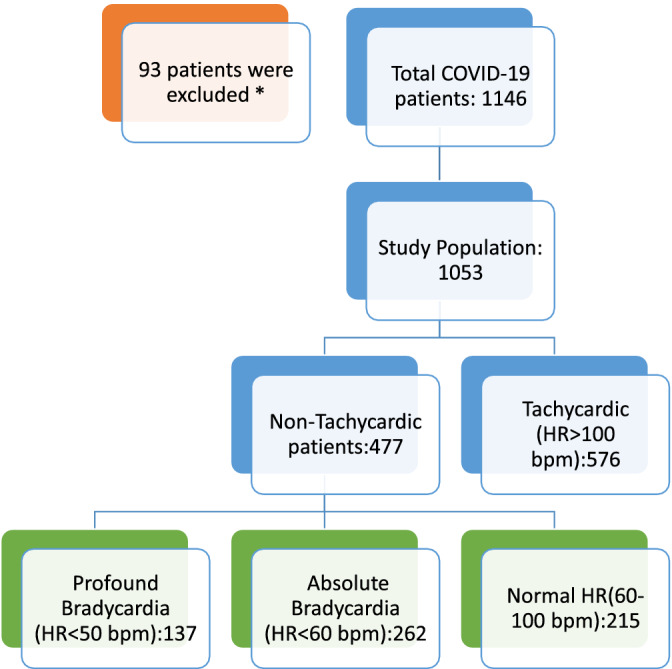
Study design. *Excluded due to end of life bradycardia, on AV nodal blockers, age < 18, or left AMA

We performed a multi‐site retrospective analysis, which included seven Southern California hospitals, on patients that had a COVID‐19 diagnosis verified by PCR from March through August 2020 (Figure 1). A total of 1143 patients were identified. Patients who were less than 18 years of age, had end of life bradycardia, were on AV nodal blocking agents, or left against medical advice were excluded from the study. Both the inclusion and exclusion criteria of a patient was verified by two physicians. A total of 1053 patients were included in final analysis. A trend of bradycardia was noted in our cohort population. Absolute bradycardia and profound bradycardia was defined as a sustained HR < 60 BPM and < 50 BPM, respectively, on two separate occasions, a minimum of 4 hours apart during hospitalization. Two physicians confirmed bradycardia events by reviewing telemetry strips as well as admission EKG to rule out any underlying arrhythmia. The oxygen saturation during the bradycardic event was noted to rule out any hypoxia‐driven bradycardia.

Chi‐square tests were performed in intergroup comparisons of categorical variables, and categorical variables were expressed as numbers, and percentages. Event rates as descriptive statistics were calculated by dividing the total number of events by total number of cases and were reported in percentages. A logistic regression analysis was performed to study the relationship between mortality and bradycardia in the study group and the effect of age, gender, race and BMI on that relationship. The effect was expressed in terms of Odds ratio with 95% CI. The calculations were performed using IBM SPSS Statistics version 27.

## 3 RESULTS

### 3.1 Baseline study characteristics

Table 1 shows the breakdown of baseline characteristics (Age, gender, and BMI) of the study population, all of which appeared to be comparable. The age ranged from 19–110 years old. The mean age ± *SD* between the bradycardia, normal, and tachycardia heart rate groups were 61.82 ± 17.01, 61.05 ± 18.96, 59.3 ± 18.41 respectively. There were 468 female patients and 585 male patients, total. Within the cohorts there were 115 females, and 147 males in the bradycardia group. In the group with a normal HR throughout admission there were 113 females, and 102 males. In the final group of patients with tachycardia there were 240 females, and 336 males. Race was evaluated and divided into white, and non‐white. There was a total of 668 white patients and a total of 385 non‐white patients. The mean BMI across all three cohorts was 30, which classically is characterized as obese. Mean ± SD admission heart rates, systolic blood pressures, and temperatures were 98.6± 28.3, 130.2 ± 25.2 , and 100.3 ±1.8 respectively for the data set.

**TABLE 1:** Baselines characteristics

		Total *N* = 1053		
		Bradycardic (*N* = 262/24.9%)	Normal HR (*N* = 215/20.4%)	Tachycardia (*N* = 576/54.7%)
		Count(%) /Mean ± *SD*	Count(%)/Mean ± *SD*	Count(%) /Mean ± *SD*
Age		61.82 ± 17.01	61.05 ± 18.96	59.32 ± 18.41
Gender	Female	115 (43.9%)	113(52.6%)	240(41.7%)
	Male	147(56.1%)	102(47.4%)	336(58.3%)
Race	Non white	96(36.6%)	76(35.3%)	213(37.0%)
	White	166(63.4%)	139(64.7%)	363(63.0%)
BMI		30.41 ± 7.93	29.41 ± 7.79	29.42 ± 7.23

In this study, there were 477 patients (45.3%) who had a non‐tachycardic response to COVID‐19. There were 262 (24.9%) verified COVID‐19 patients with absolute bradycardia, and 137 (13.0%) who had profound bradycardia. The COVID‐19 database was then separated into three cohort groups based on heart rate; bradycardia, normal heart rate, and tachycardia as defined above, to compare the findings of absolute bradycardia and normal heart rate response.

### 3.2 Associated mortality in patients with COVID‐19 and absolute bradycardia

In this study population the overall mortality, **(**Supplemental Figure 2) for patients with COVID‐19 during the months of March through August 2020 was 18.7%. In addition, 17.1% of our study population was on a ventilator. There were 856 patients that survived hospitalization from COVID‐19, while 197 expired. Patients with a HR < 50 BPM had a 25.5% mortality rate, while patients with a HR < 60 BPM had a mortality rate of 17.7% (Table 2). The logistic regression model studying the predictors of mortality was statistically significant, X^2^ (5)=47.10, p < .001. The model explained 19.6% (Nagelkerke R^2^) of variance in the mortality and correctly classified 88.6% of cases. Patients with heart rate < 60 BPM were 6.59 times (95% CI [2.83–15.36]) more likely to expire compared to the patients with HR > 60 BPM (Table 3).

**TABLE 2:** Mortality in each heart rate subgroup

			Heart rate category			
			Bradycardia	Normal HR	Tachycardia	Total
Mortality	Alive	Count	216	205	435	856
		%	25.2%	23.9%	50.8%	100.0%
	Dead	Count	46	10	141	197
		%	23.4%	5.1%	71.6%	100.0%
Total		Count	262	215	576	1053
		%	24.9%	20.4%	54.7%	100.0%

**TABLE 3:** Regression coefficients obtained from the logistic binomial regression model studying the predictors of mortality in the study group

								95% CI for EXP(B)	
		B	*SE*	Wald	df	Sig.	Exp(B)	Lower	Upper
a	Brady vs Normal (1)	1.886	0.432	19.071	1	0	6.591	2.827	15.364
	Age	0.047	0.011	16.838	1	0	1.048	1.025	1.072
	Gender	−0.157	0.323	0.235	1	0.628	0.855	0.454	1.611
	Race	−0.216	0.342	0.4	1	0.527	0.805	0.412	1.576
	BMI	0.002	0.023	0.008	1	0.929	1.002	0.959	1.047
	Constant	−6.352	1.286	24.406	1	0	0.002		

*Note*: ^a^Variable(s) entered on step 1: Brady vs Normal, Age, Gender, Race, BMI.

### 3.3 Associated mortality in patients with COVID‐19 and age > 60

The above regression revealed that for every year of age > 18, the odds of mortality due to COVID‐19 increased by 1.048 times. Table 4 shows the percentage of patients with age > 60 that expired due to COVID‐19. 547 patients (51.9%) of our study population was >60 years of age and 112 (20.4%) of those patients had an absolute bradycardic event. 38 (33.9%) of those that had an absolute bradycardic event expired. Within the population of age > 60 with an absolute bradycardic event, the odds ratio of mortality is 2.77, (95% CI [1.25–5.27]).

**TABLE 4:** Mortality rate of COVID‐19 with age ≥ 60 and age < 60

	Expired flag				Total	
	Alive		Dead			
	*N*	%	*N*	%	*N*	%
Age < 60 years	452	52.8%	54	27.4%	506	48.1%
Age ≥ 60 years	404	47.2%	143	72.6%	547	51.9%
Total	856	100.0%	197	100.0%	1053	100.0%

## 4 DISCUSSION

While cardiac manifestations are a well‐recognized complication of COVID‐19 pneumonia, bradycardia has only been described in isolated case reports,^4^, ^5^ a limited case series^6^ of relative bradycardia defined as a heart rate (HR) <90 beats per minute (BPM)^7^ and through a survey sent among cardiac electrophysiologists.^3^ This is the first study with this large of a population (*n* > 1000) that comments on the mortality and prevalence of absolute and profound bradycardia in COVID‐19 patients.

In this large multi‐center retrospective study of patients with verified COVID‐19 viral infection, absolute bradycardia (HR < 60 BPM) was seen in 24.9% of patients. Patients with bradycardia were associated with adverse outcomes, including 6.59 times greater odds of mortality compared with a normal heart rate. Tachycardia response was noted in 50.8% of the cases, which is expected in a SIRS and/or Sepsis. However, 51.3% of the patients had a non‐tachycardic response which is an unexpected finding in our study. Therefore, it is imperative to identify and triage these patients earlier as they are a high‐risk group for potentially adverse outcomes.

Amaratunga et al hypothesized that the mechanism of bradycardia could be related to the direct pathogenic effects on the myocardium, the release of inflammatory cytokines, or systemic autonomic dysregulation.^4^ Specifically, within COVID‐19 cases, higher levels of angiotensin‐converting enzyme (ACE2) are found (the surface protein which coronavirus uses to enter the cell). Conventionally, bradykinin causes hypotension and reflex tachycardia. A study with rabbits given bradykinin with an ACE inhibitor was shown to have a paradoxical bradycardic response.^8^, ^9^ It is possible that COVID‐19 may be responsible for a similar effect in humans and lead to bradycardia in our population.

In our study population, common causes of bradycardia such as hypoxia, AV‐nodal blockers, and vasovagal bradycardia were ruled out. After the end of life bradycardic events were excluded from the data, a Spearman rho's correlation was calculated to evaluate the relationship between hypoxia and bradycardia. The correlation coefficient was −0.140 with a p‐value of 0.175 which showed no statistically significant correlation between oxygen saturation and bradycardia. In addition, those that were in the profound bradycardic group had a mean oxygen saturation of 95 ± 0.04 with 19.5% of the group on a ventilator.

Each bradycardic event was also subsequently reviewed by two physicians with telemetry strips. Admission EKGs were also examined to rule out any underlying arrhythmia at admission. Patients on AV‐nodal blockers causing bradycardia were also excluded. To preclude a vaso‐vagal response causing bradycardia, the bradycardia event must have been sustained with a minimum of 4 hours apart.

Ye et al as well as Amaratunga et al hypothesized that the inflammatory cytokine storm may affect cardiac pacemaker cells leading to bradycardia.^4^, ^10^, ^14^ In our study, we tried to correlate the inflammatory markers with those that went bradycardic. Inflammatory markers such as LDH, D‐Dimer, CRP, Ferritin, and CPK were collected for our study population. However, Inflammatory markers were not ordered daily on patients, so we were unable to correlate the inflammatory markers with events of bradycardia (Supplemental Table 5).

Statistical analysis of limited data demonstrated that LDH and CRP were significantly higher in the non‐survivor versus survivor group. LDH and CRP were also significantly higher in the marked bradycardia group versus normal HR group. In the non‐survivor group D‐Dimer and CPK were also significantly higher when compared with the survivor group. This finding differs from the marked bradycardia group where it was observed that ferritin was significantly higher than in the normal HR group.

However, this is particularly interesting because recent studies have shown increased mortality closely related to cytokine storm. The inflammatory markers released could affect conduction like Ye et al hypothesized. Development of bradycardia, in the future, may be of diagnostic value for impending cytokine storm of COVID‐19 and should be looked into further.

COVID‐19 medications commonly used within a hospital setting, such as Remdesivir, Dexamethasone, and Tocilizumab, were investigated. In this study, 52 (28.7%), 26 (29.2%), and 152 (30.8%) patients who received Remdesivir, Dexamethasone, and Tocilizumab, respectively were within the group who had an absolute bradycardic response. Of note, there was high incidence of bradycardia in individuals who did not receive the medications thus we hypothesize that these medications should not be the sole cause of bradycardia.

In addition, the authors attempted to look retrospectively at EKGs or echocardiograms obtained at the time of a marked bradycardia event. Unfortunately, a complete data set could not be obtained as these patients were part of the initial COVID surge when the main focus was to limit staff exposure to COVID. Only 34 echocardiograms were obtained among individuals who had a profound bradycardia event, with a mean ejection fraction of 62% with a *SD* ± 0.08%.

There is limited data on COVID‐19 patients with bradycardia and conflicting recommendations on the management. While The European Society of Cardiology suggested an initial trial of isoprenaline and atropine, a recent Italian paper proposed an early permanent pacemaker implantation to avoid the risk of infection with a temporary pacemaker.^11^, ^12^

Our study was limited by its retrospective, cohort design. All patients with documented COVID‐19 infection from the seven southern California hospitals were initially included in our study. The data collection was dependent on the results manually entered into the EMR, which caused some discrepancies. One such discrepancy noted was the subjective reporting of race in the EMR; patients were categorized as white vs non‐white based on information entered by admission personnel, and we were unable to independently verify the accuracy of the information.

Lastly, this study can only comment on an association between bradycardia and mortality and not a causation, due to the retrospective design. In the future, the timing of the inflammatory markers with the bradycardic event, the timing of the onset of bradycardia, and COVID‐19 medications, such as Remdesivir and Dexamethasone, should be researched further to help us determine the pathophysiology of the bradycardia.

## 5 CONCLUSION

The clinical implications of this novel descriptive study include the following:

(1) This is the first large scale study to report an absolute bradycardia response in COVID‐19 patients.

(2) Within the bounds of our study, no obvious cause for bradycardia was identified in these patients suggesting a primary toxic effect of COVID‐19 infection on heart rate.

(3) Absolute bradycardia events during hospital admission were associated with an odds ratio of 6.59 for mortality as compared with patients who had a normal heart rate response, therefore we recommend early triage and appropriate level of care in this high‐risk group.

### 5.1 Clinical perspective

This retrospective cohort study reports that a quarter of hospitalized COVID‐19 patients had an absolute bradycardia event. Cases with a bradycardia event are particularly associated with higher odds of mortality compared to those with normal heart rates. There was no obvious cause of bradycardia identified in these patients, suggesting a primary toxic effect of COVID‐19 infection on heart rate. Therefore, it is imperative to identify and triage these patients earlier as they are a high‐risk group for potentially adverse outcomes. In the future, elevation of inflammatory marker in correlation with onset of bradycardia can be elucidated in detail. The impact of COVID‐19 medications, such as Remdesivir and Dexamethasone, can also be investigated further to help determine the effect on bradycardia.

## CONFLICT OF INTEREST

The author(s) declare no conflict of interest for this work.

## Supporting information


**Supplemental Figure 2** Bar Diagram of Mortality in patients with a Non‐Tachycardic Response to COVID‐19

**Supplemental Figure 3** Central illustration

**Supplemental Table 5** Inflammatory markers collected for study population

## Data Availability

The data that support the findings of this study are available from the corresponding author upon reasonable request.
