# Supplementary material for: A novel study on SARS‐COV‐2 virus associated bradycardia as a predictor of mortality‐retrospective multicenter analysis
Source: Clin Cardiol. 2021 May 8;44(6):857–62. doi: 10.1002/clc.23622 (PMC8207973; doi:10.1002/clc.23622)

Supplemental Figure 2: Bar Diagram of Mortality in patients with a Non-Tachycardic Response to COVID-19

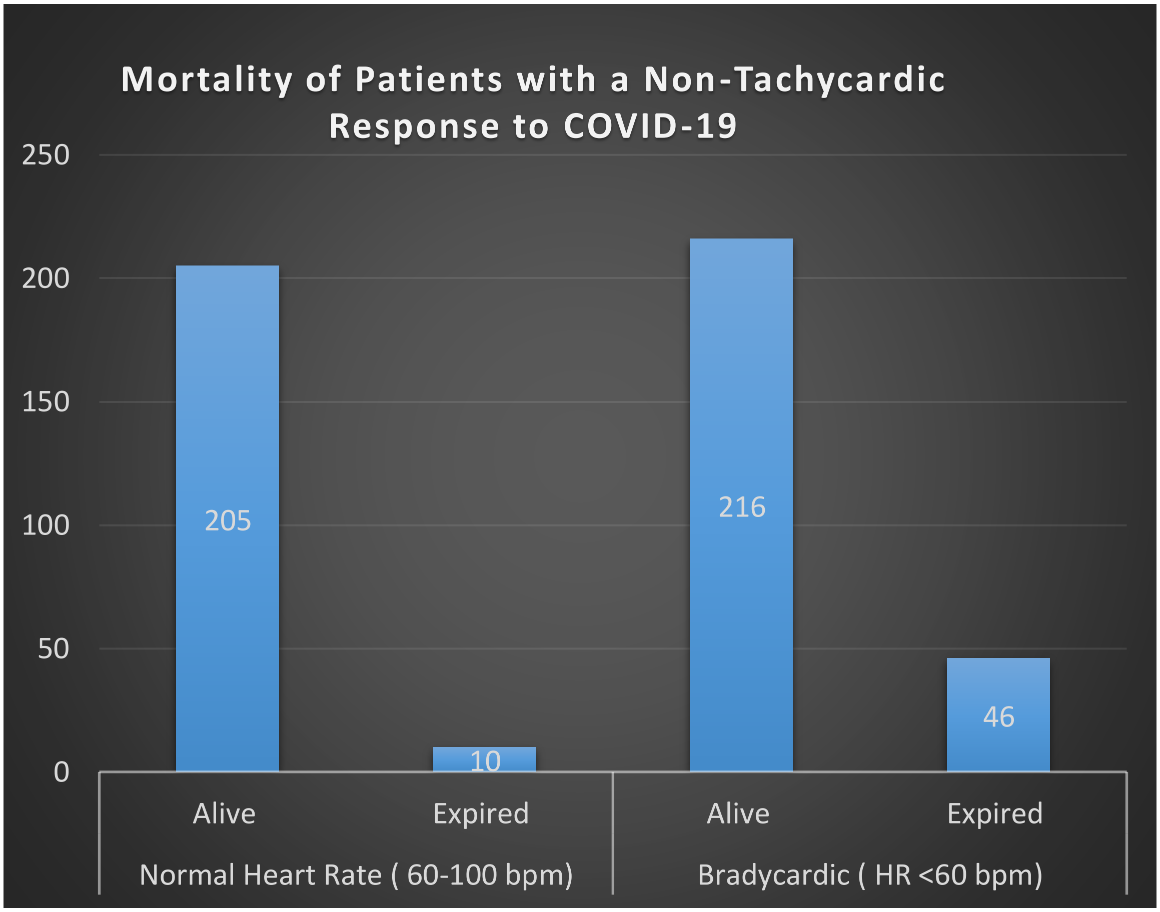

Supplement: Supplementary file 1 — Supplemental Figure 2 Bar Diagram of Mortality in patients with a Non‐Tachycardic Response to COVID‐19 [file CLC-44--s002.pdf]
