# Supplementary figures and images for: A novel study on SARS‐COV‐2 virus associated bradycardia as a predictor of mortality‐retrospective multicenter analysis
Source: Clin Cardiol. 2021 May 8;44(6):857–62. doi: 10.1002/clc.23622 (PMC8207973; doi:10.1002/clc.23622)

Figure 1: Study Design

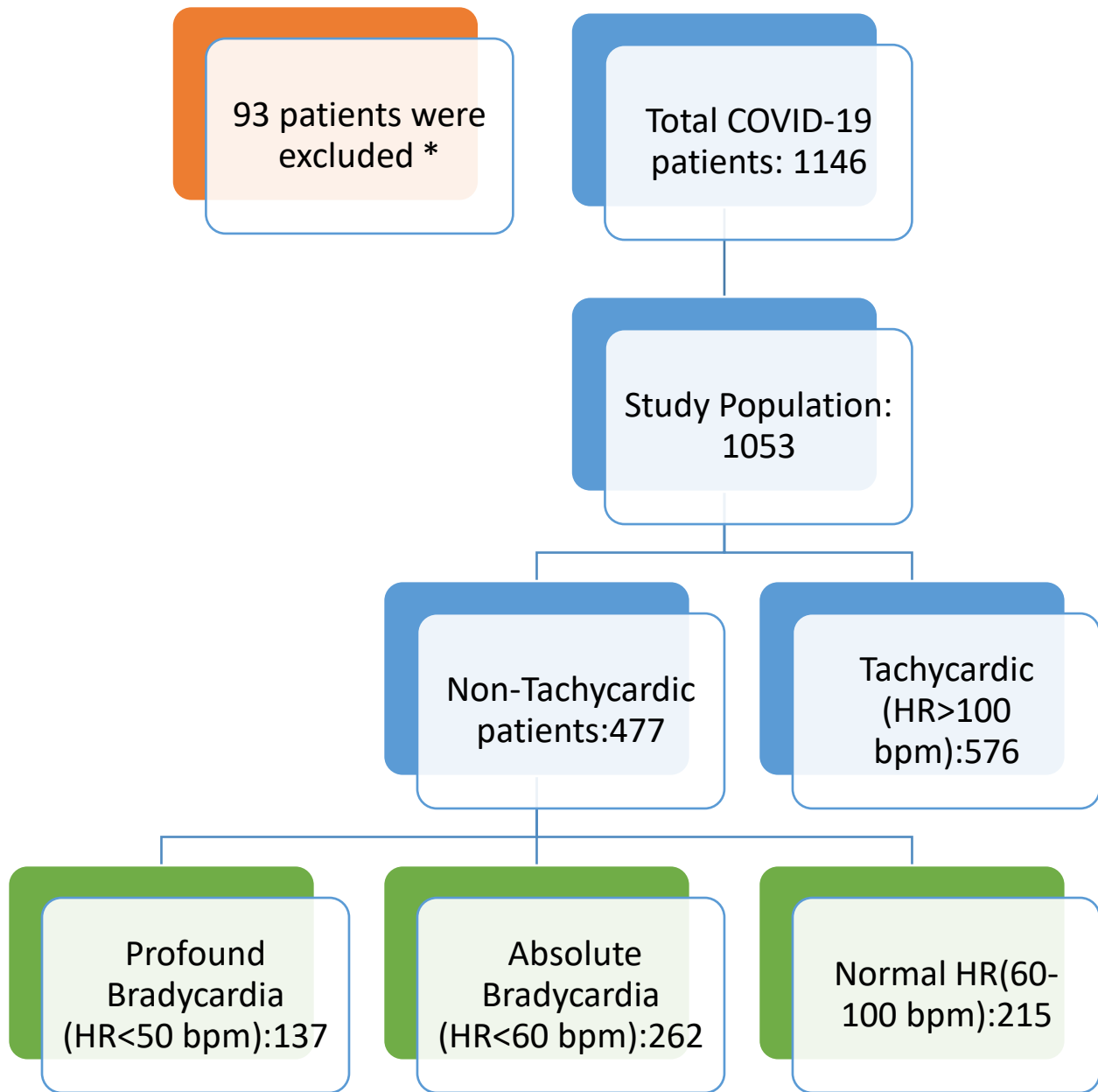

\*Excluded due to end of life bradycardia, on AV nodal blockers, age < 18, or left AMA

Supplement: Supplementary file 2 — Supplemental Figure 3 Central illustration [file CLC-44--s001.pdf]
