# Supplementary material for: A novel study on SARS‐COV‐2 virus associated bradycardia as a predictor of mortality‐retrospective multicenter analysis
Source: Clin Cardiol. 2021 May 8;44(6):857–62. doi: 10.1002/clc.23622 (PMC8207973; doi:10.1002/clc.23622)
Supplement: Supplementary file 3 — Supplemental Table 5 Inflammatory markers collected for study population [file CLC-44--s003.pdf]

Supplemental Table 5: Inflammatory markers collected for study population

	Total (N=477)	Expired (N=56)	Alive (N=421)	p-value
LDH (Mean±SD)	314.9±225.3	506.3±501.0	288.1±133.0	< 0.001
D-dimer (Mean±SD)	1097.9±1233.5	1711.9±1403.7	1007.7±1182.3	< 0.001
CRP (Mean±SD)	9.5±8.3	14.1±9.7	8.7±7.7	< 0.001
Ferritin (Mean±SD)	582.1±743.7	698.6±1092.5	564.4±676.0	0.265
CPK (Mean±SD)	471.8±2866.1	1735.2±6107.3	279.3±1912.6	0.007
